# In situ mapping of activated PDGFRβ defines a prognostic discrepancy between histological subtypes of NSCLC

**DOI:** 10.1186/s12964-026-02651-3

**Published:** 2026-01-15

**Authors:** Amanda Lindberg, Louise Hellberg, Anaïs Grandon, Hui Yu, Viktoria Thurfjell, Erik Wåhlén, Neda Hekmati, Max Backman, Axel Cederholm, Artur Mezheyeuski, Anna Klemm, Johan Botling, Agata Zieba Wicher, Patrick Micke, Carina Strell

**Affiliations:** 1 Department of Immunology, Genetics and Pathology, Uppsala University, Uppsala, Sweden; 2 Department of Pharmaceutical Biosciences, Uppsala University, Uppsala, Sweden; 3 Molecular Oncology Group, Vall d’Hebron Institute of Oncology, Barcelona, Spain; 4 Vall d’Hebron Institute of Research, Barcelona, Spain; 5 BioImage Informatics Unit, Science for Life Laboratory, Department of Information Technology, Uppsala University, Uppsala, Sweden; 6 Department of Laboratory Medicine, Institute of Biomedicine, University of Gothenburg, Gothenburg, Sweden; 7 Navinci Diagnostics AB, Uppsala, Sweden; 8 Centre for Cancer Biomarkers CCBIO, Department of Clinical Medicine, University of Bergen, Bergen, Norway

## Abstract

**Background:**

Increased stromal Platelet-derived growth factor receptor beta (PDGFRβ) expression is a hallmark of the desmoplastic tissue reaction in cancer and marks subsets of cancer-associated fibroblasts, pericytes, and smooth muscle cells. However, its functional status in situ has been anticipated from static expression measures, which cannot determine whether high receptor abundance reflects active signaling.

**Methods:**

We established two second-generation proximity ligation assays (PLAs) to quantify PDGFRβ activation in the in situ environment of human lung cancer by detecting either phosphorylated PDGFRβ or its interaction with the adaptor protein Grb2. The immunofluorescence-based assays were applied to tissue-microarrays including diagnostic samples from over 600 non-small cell lung cancer (NSCLC) patients.

**Results:**

In lung cancer tissue, activation scores correlated with PDGFRβ expression but revealed a more nuanced receptor status, indicating variable activation despite similar expression levels. Higher PDGFRβ activation was associated with increased recurrence risk exclusively in squamous cell carcinoma, a finding not captured by conventional immunohistochemistry. This activation was accompanied by a specific stromal profile enriched for LRRC15- and FAP-positive cells, a pattern absent in adenocarcinomas.

**Conclusion:**

PDGFRβ activation status provides functional information beyond receptor expression, uncovering clinically relevant, otherwise overlooked, stromal phenotypes. The approach illustrates the diagnostic potential of functional protein assays in the era of precision medicine.

**Supplementary Information:**

The online version contains supplementary material available at 10.1186/s12964-026-02651-3.

## Introduction

Platelet-derived growth factors (PDGFs) are known as pivotal signaling molecules regulating functions of mesenchymal cells, such as fibroblasts, smooth muscle cells, and pericytes, via the two PDGF-receptor isoforms (PDGFR-α and -β). PDGFRs are receptor tyrosine kinases (RTKs) that dimerize upon ligand stimulation, resulting in the autophosphorylation of several residues within the tyrosine kinase domain. A series of different intracellular signaling cascades can thereby be initiated through binding of downstream molecules, including adapter molecules such as Growth factor receptor-bound protein 2 (Grb2), to the phosphorylated tyrosines [1–6]. In cancer biology, the PDGF-signaling axis is involved in reciprocal communication between tumor and stromal cells, influencing cancer progression and therapy outcome [7].

In preclinical models, PDGFRβ signaling has been linked to increased tumor growth, metastasis, and increased interstitial fluid pressure, contributing to resistance against various treatments [7, 8]. Furthermore, high stromal expression of PDGFRβ is associated with poor prognosis in several human solid tumors, including breast, prostate, and liver cancers [9–12]. Given these findings, tyrosine kinase inhibitors (TKIs) targeting PDGFRs have been tested in multiple cancer types with varying results. Indeed, TKIs such as Sunitinib and Imatinib exhibit anti-tumorigenic effects and are approved for the treatment of leukemia, renal cell carcinoma, and gastrointestinal stromal tumors [13–15]. However, there are no PDGFR-specific inhibitors, and currently available ones block multiple kinases simultaneously (including e.g., Vascular endothelial growth factor [VEGFR] and c-Kit) with similar affinities. Therefore, it is difficult to attribute any observed effects specifically to the inhibition of PDGFR signaling. Furthermore, these multikinase inhibitors have failed to show relevant effects in breast, pancreatic, prostate, and lung cancers [16–20]. Another explanation for the different responses of these targeted approaches could be the varying expression levels of PDGFRβ and the biological impact of the signaling pathway in the complex cellular networks of cancer. Notably, while many pro-tumorigenic effects of PDGFR signaling are linked to cancer-associated fibroblasts (CAFs), specific depletion of PDGFRβ in pericytes has been found to enhance cancer metastasis, underscoring both the potential and the complexity of PDGFRβ signaling as a therapeutic target [7, 8].

To capture the heterogeneity of PDGFRβ expression in cancer tissue, most previous studies have relied on immunohistochemistry (IHC) or immunofluorescence. Such studies have indicated high variability and heterogeneity in PDGFR expression, with some tumors exhibiting a high and diffuse stromal expression pattern, while others lack PDGFRβ expression [9, 10, 21–23]. However, protein expression alone does not reflect receptor activation and the subsequent biological impact. Conceptually, it would be better to directly measure receptor activation to obtain the functional information from patient tissues. Therefore, researchers often use phospho-specific antibodies to obtain the activation status of a specific pathway; however, these antibodies frequently lack sufficient specificity for the IHC application in FFPE tissues, making reliable in situ stainings challenging. This is due to the fact that the phosphorylation sites within the intracellular kinase domain of PDGFRβ are highly homologous amongst RTKs, particularly with PDGFRα, leading to potential cross-reactivity and unspecific staining signals [24]. Furthermore, PDGFRβ has several tyrosine residues [25], each associated with specific downstream signaling [1–5], making single-residue phosphorylation assessments potentially insufficient to capture full receptor activation. Finally, receptor phosphorylation is transient, probably short-lived and unstable, and might not be preserved during the slow formalin penetration during the fixation process [26].

With this background, we adapted a second-generation proximity ligation assay (PLA) to investigate PDGFRβ activation in the stromal compartment of non-small cell lung cancer (NSCLC), since a reactive stroma is a hallmark of NSCLC and mediates the desmoplastic process. Previous studies assessing stromal PDGFRβ in NSCLC have reported no or inconclusive prognostic impacts based on expression levels via IHC or immunofluorescence [21, 22, 27]. In this study, we used the PLA workflow to quantify stromal PDGFRβ activation in situ by detecting its interaction with the adaptor protein Grb2, and benchmarked this against phosphorylated PDGFRβ (phosphoPDGFRβ) using the same assay. We then applied this approach to diagnostic tissue samples from a well-characterized cohort of NSCLC patients.

## Materials and methods

### Patient cohort

The basis of the study was the combination of two extensively characterized patient cohorts consisting of surgically treated NSCLC patients treated at Uppsala University Hospital (Uppsala Akademiska Sjukhus) between 1995 and 2010. The cohort originally consisted of 679 patients, of which 601 had enough viable tissue left for the analysis of Grb2-PDGFRβ analysis (537 for the phosphoPDGFRβ analysis).

Tissue microarrays (TMAs) were constructed from formalin-fixed paraffin-embedded (FFPE) tumor tissue blocks with two 1 mm cores from representative tumor areas from each patient as described previously [28, 29]. In a few cases, two primary tumors from the same patient were available, in which case both were included. RNAseq data was available for a subset of patients obtained previously with the standard Illumina RNAseq protocol in the HiSeq2500 instruments (Illumina) as described in [30]. The raw data are available via GEO, with the accession number GSE81089 (http://www.ncbi.nlm.nih.gov/geo/). This study was conducted in accordance with the Declaration of Helsinki and the Swedish Ethical Review Act (Approvals #2006/325, #2012/532). A control TMA constructed from a small subset of cases was used for antibody control experiments.

### Tissue preparation and PLA staining

Four-micron-thick FFPE sections were mounted onto adhesive slides and dried at 50 °C overnight. Deparaffinization, antigen retrieval, and Pan-Cytokeratin (CK) staining were performed with an automated protocol using Bond RX_m_ (Leica Biosystems, Nussloch, Germany) with bulk reagents from Leica. Deparaffinization was performed with Dewax solution (#AR9222) followed by antigen retrieval with Epitope retrieval solution 2 (ER2, pH 9, #AR9640) at 100 °C for 30 min. The cytokeratin staining was preceded by Blocking buffer (Navinci Diagnostics, Uppsala, Sweden) incubation for 5 min. Further, the primary antibody incubation was performed with an anti-panCK antibody (AE1/AE3, Agilent Dako, Santa Clara, CA, USA) diluted in Dako Real Antibody Diluent (#S2022) 1:400 for 30 min. A secondary ImmPress-mouse HRP antibody (MP-7402, Vector Laboratories, Newark, CA, USA) was added for 10 min, followed by a tyramide signal amplification step with Opal 520 (#FP1487001KT, Akoya Biosciences, Marlborough, MA, USA), diluted 1:150 in 1X Plus Manual Amplification Diluent (#FP1135, Akoya) for 10 min. A second antigen retrieval step was performed with ER1 solution (ER1, pH6, #AR9961) at 100 °C for 20 min. All steps listed had subsequent washing steps performed with Wash solution (#AR9590).

From this step forward, manual staining was performed with reagents from Navinci Diagnostics to perform second-generation PLAs [31]. The Grb2-PDGFRβ assay was performed using a predecessor version of the NaveniBright kit (#NB.MR.HRP.100) using HRP-conjugated oligo detection probes. The PLA was conducted according to the manufacturer’s instructions up until the addition of Opal 620 instead of HRP-substrates (#FP1495001KT, Akoya) diluted 1:150 for a fluorescent readout. Spectral DAPI (#FP1490, Akoya) was added as a counterstain. Primary antibodies used were anti-PDGFRβ (clone: 28E1, Cell Signaling Technology^®^, Danvers, MA, USA) diluted 1:150 and anti-Grb2 (clone: 81, BD Transduction Laboratories, Franklin Lakes, NJ, USA) 1:7000. The phosphoPDGFRβ assay was performed using an earlier beta-version of the NaveniFlex kit (#NT.MR.100:RED, Navinci) with customary detection oligos without fluorochromes and conjugated HRP instead. Primary antibodies used were the anti-PDGFRβ (28E1) diluted 1:100 and an anti-phospho-tyrosine (#P-Tyr-100, Cell Signaling) diluted 1:1600, and the readout reagents were the same Opal and DAPI dilutions as for the Grb2 assay.

Briefly described, blocking buffer was applied to the sample areas and incubated at 37 °C for 1 h in a humidity chamber. Primary antibody incubation was performed at 4 °C overnight. For the antibody control experiments, only one or no antibody was used. Secondary antibodies (Probes/Navenibodies) anti-mouse and anti-rabbit were each diluted 1:40 in Probe diluent and incubated at 37 °C for 1 h, followed by a washing step. Reaction 1 was added to the slides and incubated at 37 °C for 30 min, followed by Reaction 2 at 37 °C for 90 min. For signal detection, HRP reagent was added to the slides and incubated at RT for 30 min, followed by Opal 620 incubation for 10 min at RT and DAPI for 5 min at RT. Slides were mounted with ProLong™ Gold Antifade Mountant (Invitrogen™, Thermo Fisher Scientific, Waltham, MA, USA).

### Fluorescent image acquisition and processing

**Fig. 1:**
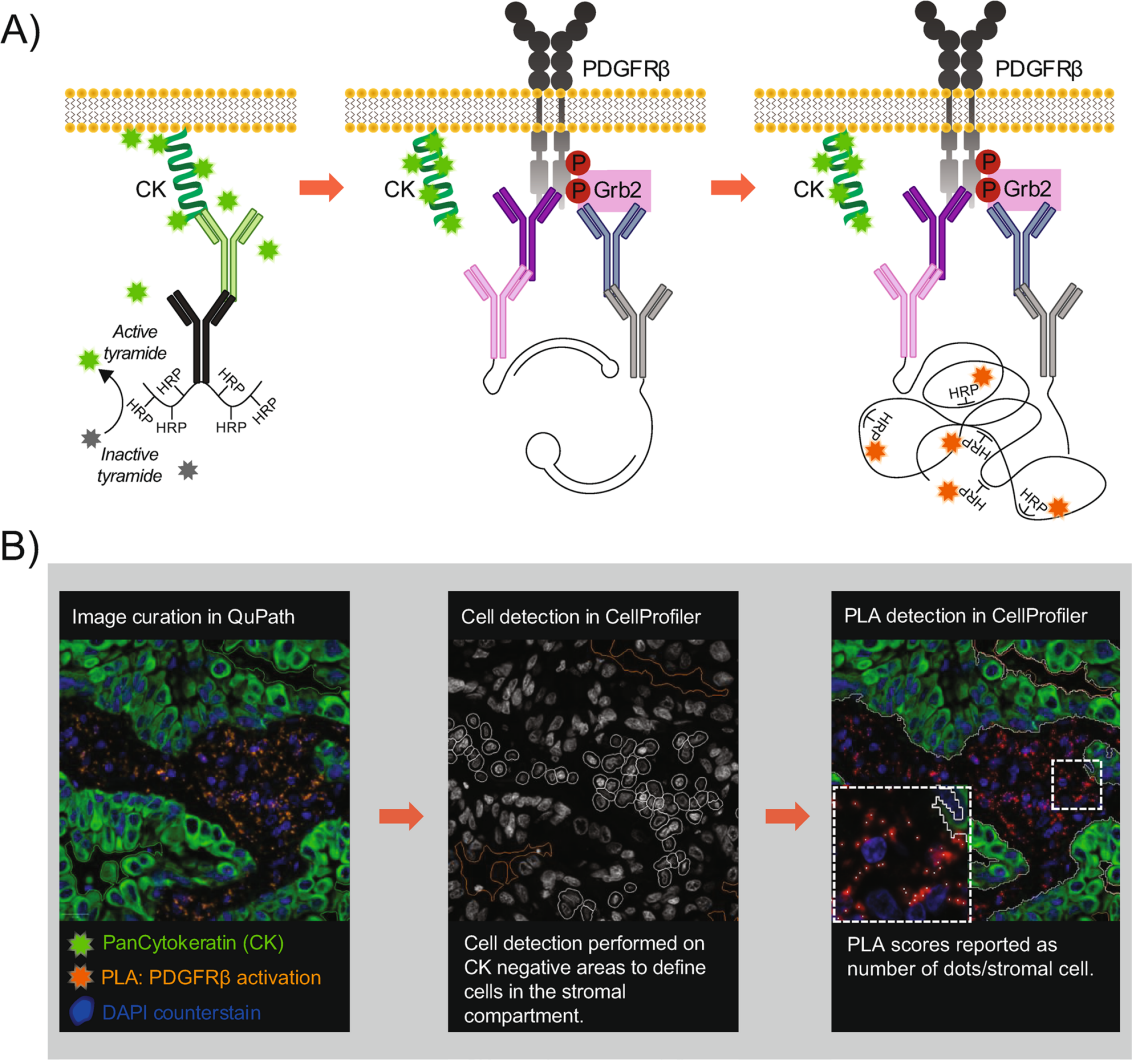
Flowchart of staining procedure and analysis. **A** Pancytokeratin (CK) staining is performed first using an anti-pan cytokeratin antibody, followed by a Horseradish peroxidase (HRP)-conjugated secondary antibody. Fluorophore-labeled tyramides (lime) are enzymatically activated by HRP and covalently bind to tyrosine residues in close proximity to the target antigen. As tyramide-tyrosine binding is covalent, all antibodies can subsequently be removed by an antigen retrieval step. Next, two primary antibodies are added: a rabbit anti-PDGFRβ and a mouse anti-Grb2 or anti-phospho-tyrosines. Species-specific secondary antibodies, each conjugated to a specific UnFold probe, are then added. UnFold probes are enzymatically processed by uracil excision, enabling probe circularization through hybridization into a DNA circle if in close enough proximity (≤ 40 nm). The DNA circle serves as a template for rolling circle amplification, generating a repetitive single-stranded DNA product, to which HRP-conjugated detection oligos can bind. A second fluorophore-labeled tyramide (orange) can then be activated and bind to tyrosine residues. Nuclei are counterstained using DAPI. **B** Image curation is performed in QuPath, removing unwanted tissue areas with potential staining artefacts prior to downstream analysis. The resulting curation masks are imported into CellProfiler, where tumor and stroma compartments are outlined based on the CK staining. Cells are segmented in the stromal (CK-negative) compartment, followed by dot detection for PLA signals (red channel). The detected PLA dots are assigned to individual stroma cells, and the PDGFRβ activation score is calculated as the average number of dots per stromal cell within each patient

The slides were scanned using the VectraPolaris™ system (Akoya Biosciences) at a resolution of 0.25 μm/pixel. Manual image curation was performed in QuPath [32] (version 0.2.3) to exclude benign lung tissue, non-tissue areas, staining artifacts, and necrotic areas. Curation masks were then imported into CellProfiler [33] (version 4.2.1). In CellProfiler, tumor compartments were segmented using a global Minimum cross-entropy (McE) threshold. The inverse of the cytokeratin-positive area defines the stroma compartment. Nuclei were then segmented using a McE thresholding for the DAPI channel and then expanded by 10 pixels to create cells. Cytokeratin-positive tumor cells were excluded from the analysis, so that only cytokeratin-negative stromal cells were retained for scoring. PLA signals were quantified using a manual global threshold. The manual threshold was tuned on several samples and then kept constant for all samples analyzed in the study. The number of PLA dots/stromal cell was extracted using the “RelateObjects” module and was averaged for all cores from the same patient (and further multiplied by 10 for the phosphoPDFGRβ assay), referred to as the ‘PLA score’. The workflow of the PLA staining procedure and its chemistry, and subsequent image processing is visually described in Fig. 1.

### IHC

PDGFRβ IHC was performed in a Bond RX_m_ (Leica Biosystems) with an initial deparaffinization step using Dewax solution (#AR9222) followed by an antigen retrieval step with ER2 solution (pH 9, #AR9640) at 100 °C for 40 min. PDGFRβ staining was performed with a Bond Polymer Refine Detection-kit (#DS9800, Leica Biosystems) with the same anti-PDGFRβ diluted 1:100. The kit applies Hematoxylin II as a counter-stain. The slides were scanned using a Nanozoomer S60 whole slide scanner (Hamamatsu Photonics, Hamamatsu City, Japan) at a resolution of 0.46 μm/pixel. The stromal expression of PDGFRβ, as a percentage of positive cells, was scored as follows according to an increasing scale: 1 = 0–1% of the stromal cells, 2 = 2–10%, 3 = 11–20%, 4 = 21–30%, 5 = 31–40%, 6 = 41–50%, 7 = 51–75%, 8 ≥ 75%. Additionally, the intensity was examined, 1 = weak, 2 = moderate, 3 = strong. The expression- and intensity-scores were then added up, creating an IHC score with a range of 2–11. Scores from cores of the same patient were averaged.

IHC stainings and annotations had been performed as previously described for the immune markers [34–36]. IHC staining of mesenchymal cells was performed in Leica Bond. Fibroblast activation protein (FAP) was stained using an anti-FAP (clone: E1V9V, Cell Signaling Technology) diluted 1:750, Leucine rich repeat containing 15 (LRRC15) using an anti-LRRC15 (clone: EPR8188[2], Abcam, Cambridge, United Kingdom) diluted 1:5000, Platelet/endothelial cell adhesion molecule-1 (PECAM-1/CD31) using an anti-CD31 (clone JC70A; Dako) diluted 1:150. The stromal expressions were scored according to the same semiquantitative scale as for PDGFRβ, without an intensity score. Scores from cores of the same patient were averaged.

### Cell culturing and fixation

BJ-hTERT fibroblasts were cultivated in F12 (Sigma-Aldrich, Merck, Darmstadt, Germany) media containing 10% fetal calf serum (Sigma-Aldrich), 1% glutamine, and antibiotics (penicillin, 100 units/ml, Sigma-Aldrich) and streptomycin (100 g/ml, Sigma-Aldrich). The cells were starved overnight in 1% fetal calf serum medium prior to stimulation with 0 ng/ml (PDGF-BB (-)) or 100 ng/ml PDGF-BB (Peprotech, Thermo Fisher) on ice for 15 min (PDGF-BB (+)). Cells were then washed in cold phosphate-buffered saline, removed from the plate, and centrifuged at 2000 rpm for 10 min. The phosphate-buffered saline was discarded, and cell pellets were incubated in a 4% phosphate-buffered paraformaldehyde solution overnight. The pellet was placed in a tissue embedding box in 70% ethanol and then placed in higher grades of alcohol for dehydration before being embedded in paraffin, sectioned, and put on Superfrost Plus slides.

### Statistical analyses

Statistical analyses were performed in SPSS (version 27.0.1.0), GraphPad Prism (version 9.4.1), and RStudio (version 2022.02.3). Dichotomization into low and high groups was predefined according to a subgroup-specific median cut-off. Contingency tables for the association of high/low groups and clinicopathological characteristics were evaluated by Fisher’s Exact tests or Fisher-Freeman-Halton Exact tests. Overall survival analysis truncated at 5 years, was undertaken using Kaplan Meier plots with Log-Rank test. Recurrence data was available for 464 patients annotated with the Grb2-PDGFRβ activation assay and 408 patients annotated with the phosphoPDGFRβ activation assay. Risk of recurrence within 5 years was assessed using cumulative hazard plots with Log-rank tests, as well as uni- and multivariable cause-specific Cox regression models in which recurrence was treated as the event and deaths without prior recurrence were censored. All p-values are two-sided and p-values < 0.05 were considered statistically significant.

## Results

### Assessment of the PLA to detect stromal PDGFRβ activation in situ

As a readout to detect PDGFRβ activation in diagnostic tissue samples, we applied two separate PLAs with two antibody pairs: either (1) the anti-PDGFRβ combined with an anti-phosphotyrosine antibody (phosphoPDGFRβ PLA) or (2) anti-PDGFRβ combined with an anti-Grb2 antibody (Grb2-PDGFRβ PLA). The Opal tyramide signal amplification system was used to combine the PLAs with a pan-cytokeratin staining step to delineate the tumor and stroma compartments (see methodology in Fig. 1A and Supplementary Fig. 1). The test staining on FFPE NSCLC tissue demonstrated intense stroma signals with both PLAs (phosphoPDGFRβ and Grb2-PDGFRβ), indicating PDGFRβ activation. The signals were quantified using a digital image analysis pipeline to calculate the mean number of PLA signals per stroma cell (PLA score) (Fig. 1B).

**Fig. 2:**
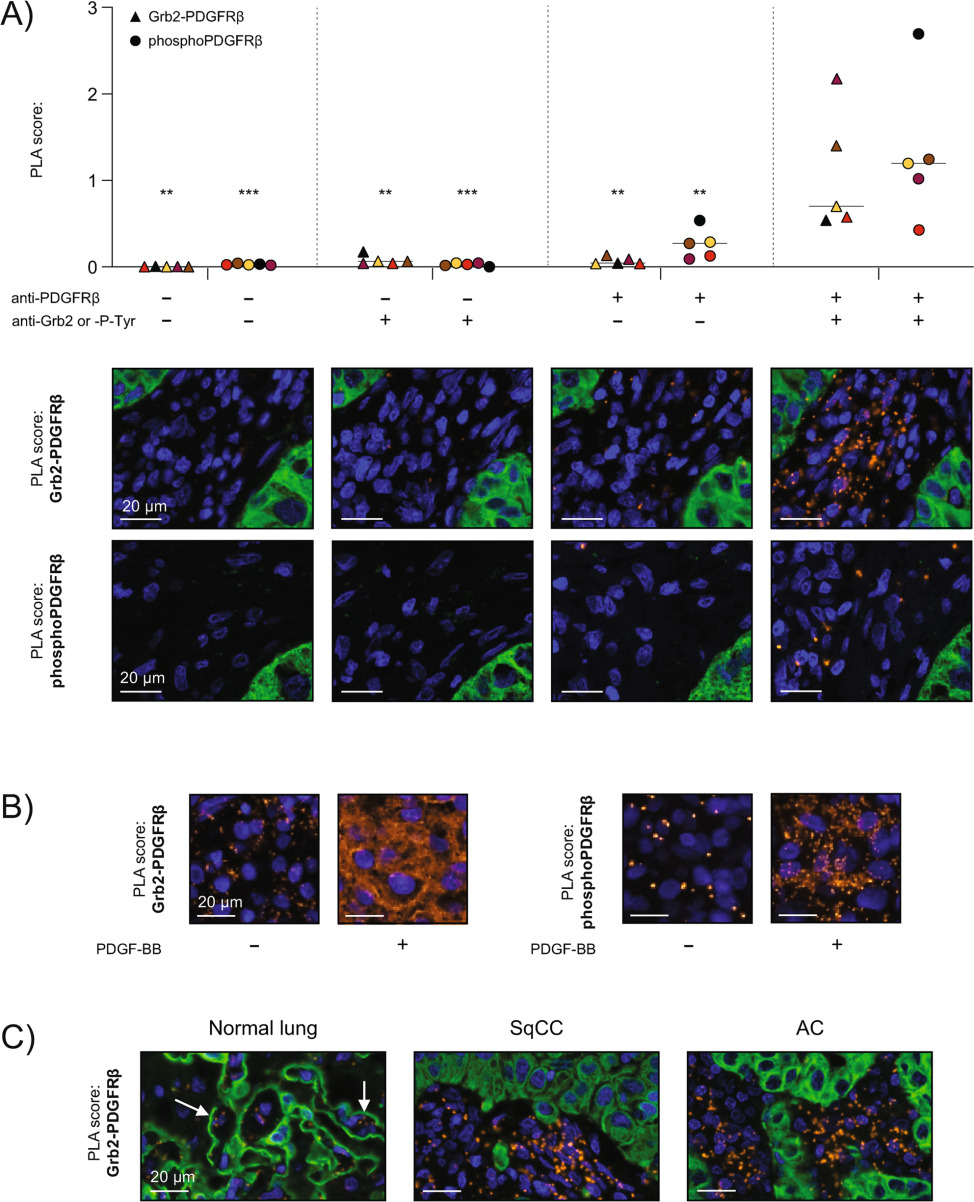
Control experiments. **A** Negative control stainings performed using either no primary antibodies, a single primary antibody, or both primary antibodies for the respective PDGFRβ activation PLAs: Grb2-PDGFRβ (triangles) and phosphoPDGFRβ (circles; illustrated as x10). Stainings were performed and analyzed on five cores from different NSCLC patients, and representative example images are shown below the graphs. Statistical analyses versus the complete PLAs (using both primary antibodies) were performed using Dunnett’s multiple comparisons test. ^**^ indicates p-values < 0.01 and ^***^ indicates p-values < 0.001. **B** Positive control experiments on PDGF-BB-stimulated human BJ-hTERT fibroblasts. Representative images of the Grb2-PDGFRβ (left) and phosphoPDGFRβ (right) PLAs are shown. Each assay was performed in triplicates. **C** Representative staining patterns in normal lung tissue, squamous cell carcinoma (SqCC), and adenocarcinoma (AC). White arrows indicate alveolar walls

To assess the specificity of the PLAs, we performed antibody control stainings on a set of consecutive NSCLC FFPE sections. PLA experiments using both primary antibodies (anti-Grb2 or anti-P-Tyr in combination with anti-PDGFRβ) demonstrate much higher PLA scores compared to PLAs excluding one or both primary antibodies, indicating a high specificity of our assay (Fig. 2A). Of note, the Grb2-assay generated on average more signals/stromal cell (1.08+/-0.71) than the phosphoPDGFRβ PLA (0.13+/-0.08, illustrated as x10 in graphs).

As an additional validation, we stimulated BJ-hTERT fibroblasts with PDGF-BB. The unstimulated FFPE-cell pellets demonstrated low PLA signal counts, whereas PDGFR activation led to diffuse and confluent PLA signals in PDGF-BB-stimulated BJ-hTERT (Fig. 2B).

Together, our control experiments suggest that our PLA methodology specifically detects and spatially maps active PDGFRβ signaling in FFPE sections via the Grb2-PDGFRβ interaction or the phosphorylation status of PDGFRβ.

### Stromal PDGFRβ activation pattern in normal lung and histological subtypes of lung cancer

Both PLAs were applied to quantify PDGFRβ activation in tissues from NSCLC patients; clinicopathological characteristics for the included patients are presented in Table 1. First, in non-malignant lung parenchyma distant to the primary lung tumor, PLA signals were observed in the alveolar wall (Fig. 2C, white arrows). In cancer tissue, generally higher PDGFRβ PLA signals were observed within the stroma, yet with a heterogeneous pattern. Squamous cell carcinoma (SqCC) and adenocarcinoma (AC) tissues demonstrated comparable levels of PDGFRβ activation scores (Table 2).

**Table 1:** Clinicopathological characteristics of the Uppsala cohort of surgically treated NSCLC patients, successfully stained and analyzed for the Grb2-PDGFRβ activation assay

Uppsala surgically treated cohort *N* = 601 (Valid %)	
Histology	
SqCC	195 (32.4)
AC	356 (59.2)
Other	50 (8.3)
Stage 8th edition	
I	294 (48.9)
II	161 (26.8)
III	138 (23.0)
IV	8 (1.3)
Age at diagnosis:	
< 70 years	365 (60.7)
≥ 70 years	236 (39.3)
Performance status (ECOG):	
0	339 (56.4)
1	232 (38.6)
2–4	30 (5.0)
Biological sex:	
Male	310 (51.6)
Female	291 (48.4)
Smoking history ^a^:	
Current/former smoker	536 (89.3)
Never-smoker	64 (10.7)
*KRAS*^a^:	
WT	435 (72.5)
Mutated	165 (27.5)
*EGFR*^a^:	
WT	539 (89.8)
Mutated	61 (10.2)
*TP53*^b^:	
WT	140 (37.0)
Mutated	238 (63.0)

*SqCC* Squamous cell carcinoma, *AC* Adenocarcinoma, *ECOG* Eastern Cooperative Oncology Group, *KRAS* Kirsten rat sarcoma virus oncogene, *EGFR* Epidermal growth factor receptor, *TP53* Cellular Tumor Antigen p53, *WT*  Wildtype (genotype)

^a^ data missing for 1 patient

^b^ data missing for 223 patients

**Table 2:** Comparison of the distribution of clinicopathological characteristics between the Grb2-PDGFRβ PLA LOW and PLA HIGH groups. P-values are based on Fisher’s exact tests for comparisons between 2 variables, and Fisher-Freeman-Halton exact tests for comparisons between > 2 variables. All tests were 2-sided. Bold values indicate statistical significance

*n* (Valid %)										
	All NSCLC patients *n* = 601				SqCC patients only *n* = 195			AC patients only *n* = 356		
Variable	PLA LOW *n* = 301 (50.1)		PLA HIGH *n* = 300 (50.1)	*p*-value	PLA LOW *n* = 98 (50.3)	PLA HIGH *n* = 97 (49.7)	*p*-value	PLA LOW *n* = 178 (50.0)	PLA HIGH *n* = 178 (50.0)	*p*-value
Histology:										
SqCC	91 (30.2)		104 (34.7)	0.477	-	-	-	-	-	-
AC	183 (60.8)		173 (57.7)		-	-		-	-	
Other	27 (9.0)		23 (7.7)		-	-		-	-	
Stage 8th edition:										
I	148 (49.2)		146 (48.7)	0.398	40 (40.8)	39 (40.2)	0.336	97 (54.5)	96 (53.9)	0.306
II	75 (24.9)		86 (28.7)		29 (29.6)	37 (38.1)		37 (20.8)	41 (23.0)	
III	72 (23.9)		66 (22.0)		29 (29.6)	21 (21.6)		38 (21.3)	40 (22.5)	
IV	6 (2.0)		2 (0.7)		0 (0.0)	0 (0.0)		6 (3.4)	1 (0.6)	
Age at diagnosis:										
< 70 years	178 (59.1)		187 (62.3)	0.452	54 (55.1)	60 (61.9)	0.384	106 (59.6)	111 (62.4)	0.664
≥ 70 years	123 (40.9)		113 (37.7)		44 (44.9)	37 (38.1)		72 (40.4)	67 (37.6)	
Biological sex:										
Male	155 (51.5)		155 (51.7)	1.000	59 (60.2)	65 (67.0)	0.373	32 (50.8)	4 (40.0)	0.736
Female	146 (48.5)		145 (48.3)		39 (39.8)	32 (33.0)		31 (49.2)	6 (60.0)	
Smoking history ^a^:										
Current/former smoker	276 (92.0)		260 (86.7)	**0.047**	95 (96.9)	88 (90.7)	0.082	159 (89.8)	150 (84.3)	0.154
Never smoker	24 (8.0)		40 (13.3)		3 (3.1)	9 (9.3)		18 (10.2)	28 (15.7)	
Performance status ECOG:										
0	163 (54.2)		176 (58.7)	0.295	47 (48.0)	49 (50.5)	0.842	106 (59.6)	112 (62.9)	0.659
1	125 (41.5)		107 (35.7)		46 (46.9)	42 (43.3)		65 (36.5)	57 (32.0)	
2–4	13 (4.3)		17 (5.6)		5 (5.1)	6 (6.2)		7 (3.9)	9 (5.1)	
*KRAS*^a^:										
WT	222 (74.0)		213 (71.0)	0.465	94 (95.9)	91 (93.8)	0.537	110 (62.1)	102 (57.3)	0.387
Mutated	78 (26.0)		87 (29.0)		4 (4.1)	6 (6.2)		67 (37.9)	76 (42.7)	
*EGFR*^a^:										
WT	264 (88.0)		275 (91.7)	0.176	96 (98.0)	97 (100.0)	0.497	147 (83.1)	152 (85.4)	0.563
Mutated	36 (12.0)		25 (8.3)		2 (2.0)	0 (0.0)		30 (16.9)	26 (14.6)	
*TP53*^b^:										
WT	69 (37.1)		71 (37.0)	1.000	9 (15.5)	7 (11.7)	0.599	54 (48.6)	63 (52.9)	0.598
Mutated	117 (62.9)		121 (63.0)		49 (84.5)	53 (88.3)		57 (51.4)	56 (47.1)	

*SqCC* Squamous cell carcinoma, *AC* Adenocarcinoma, *ECOG* Eastern Cooperative Oncology Group, *KRAS* Kirsten rat sarcoma virus oncogene, *EGFR* Epidermal growth factor receptor, *TP53* Cellular Tumor Antigen p53, *WT* Wildtype (genotype)

^a^ data missing for 1 patient

^b^ data missing for 223 patients

### Association between stromal activation status and levels of PDGF receptor and ligands

**Fig. 3:**
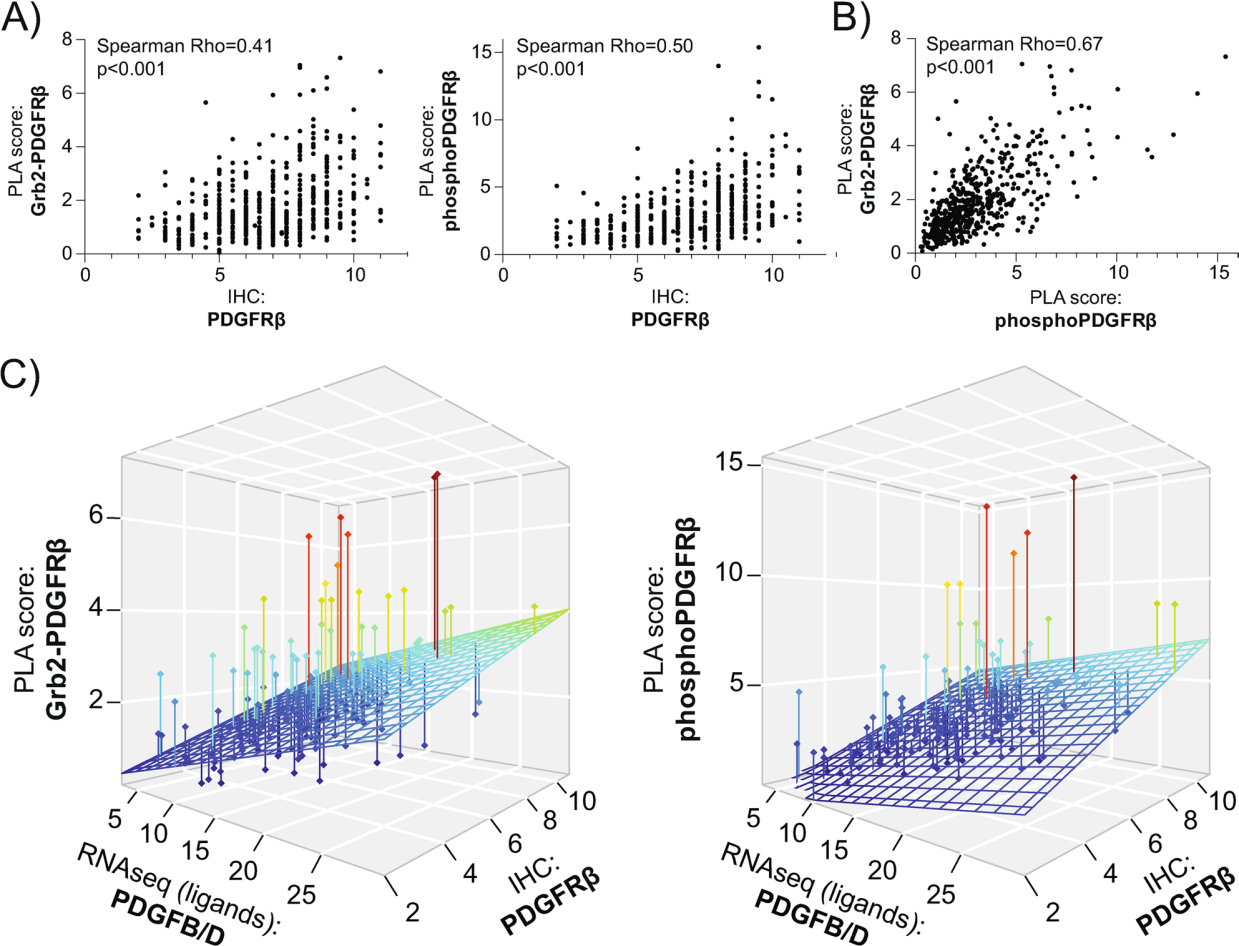
PDGFRβ activation scores correlate with ligand and receptor expression. **A** Correlation between the Grb2-PDGFRβ (left) or phosphoPDGFRβ (right) PLA activation scores and general PDGFRβ protein expression assessed via immunohistochemistry (IHC). Spearman’s rank correlation coefficients (Rho) with two-tailed p-values are indicated. **B** Correlation between Grb2-PDGFRβ and phosphoPDGFRβ activation scores. Spearman’s rank correlation coefficient (Rho) with two-tailed p-value is indicated. **C** 3D-plots illustrating the relationship between the Grb2-PDGFRβ (left) or the phosphoPDGFRβ (right) PLA scores, *PDGFB/D* gene expression levels, and PDGFRβ protein expression assessed via IHC

To assess how general PDGFRβ protein levels correlate with the stromal PDGFRβ activation scores (mean signal number/stromal cell) we performed an immunohistochemical staining of the TMA. The total amount of stromal PDGFRβ protein expression revealed a moderate correlation to the activation score quantified with both assays (Spearman correlation, Rho = 0.41, *p* < 0.001 for the Grb2-PDGFRβ assay, and Rho = 0.50, *p* < 0.001 for the phosphoPDGFRβ assay) (Fig. 3A). Importantly, these analyses demonstrated that a high PDGFRβ PLA virtually never occurred without measurable PDGFRβ protein expression. Further, a Spearman correlation investigating the relationship of the two PLAs indicated a strong correlation between the Grb2-PDGFRβ and the phosphoPDGFRβ assay, indicating that either assay could serve as a surrogate for the other (Rho = 0.67, *p* < 0.001, Fig. 3B).

To assess the dependency of stromal PDGFRβ activation on receptor and ligand levels, stromal PDGFRβ activation scores were correlated with the PDGFRβ protein expression based on IHC, as well as with mRNA expression levels of the ligands PDGF-BB and PDGF-DD, as based on RNAseq (*PDGFB*, *PDGFD*). The F-test statistics demonstrated an adjusted R^2^ = 0.17, *p* < 0.001 for the Grb2-PDGFRβ assay, and adjusted R^2^ = 0.22, *p* < 0.001 for the phosphoPDGFRβ assay, indicating moderate positive correlations between PLA scores and the combined presence of both high PDGFRβ and ligand levels (Fig.3C). Taken together, these correlative data between activation status, receptor, and ligand levels underscore the biological significance of PLA-based readout for PDGFRβ activation.

Since the Grb2-PDGFRβ and the phosphoPDGFRβ PLA strongly correlated (Fig. 3B) and showed comparable signal frequency distributions (Supplementary Fig. 2), we focused our subsequent statistical analysis on the Grb2-PDGFRβ score, dichotomized using subgroup-specific median cut-offs for the entire cohort, SqCC, and AC patients, respectively (Supplementary Fig. 2 + 3).

### Stromal PDGFRβ activation status and clinical outcome

In our NSCLC cohort, clinicopathological features, including histology, stage, age, sex, performance status, and *KRAS*, *EGFR*, and *TP53* mutation statuses, were balanced between patients within the Grb2-PDGFRβ PLA low and high groups. The only variable that differed was smoking history, where never-smokers were significantly enriched in the high PDGFRβ activation group (median cut-off, *p* = 0.047, Fisher’s exact test; Table 2). No variables differed when the cohort was stratified by the phosphoPDGFRβ PLA score instead (Supplementary Table 1).

**Fig. 4:**
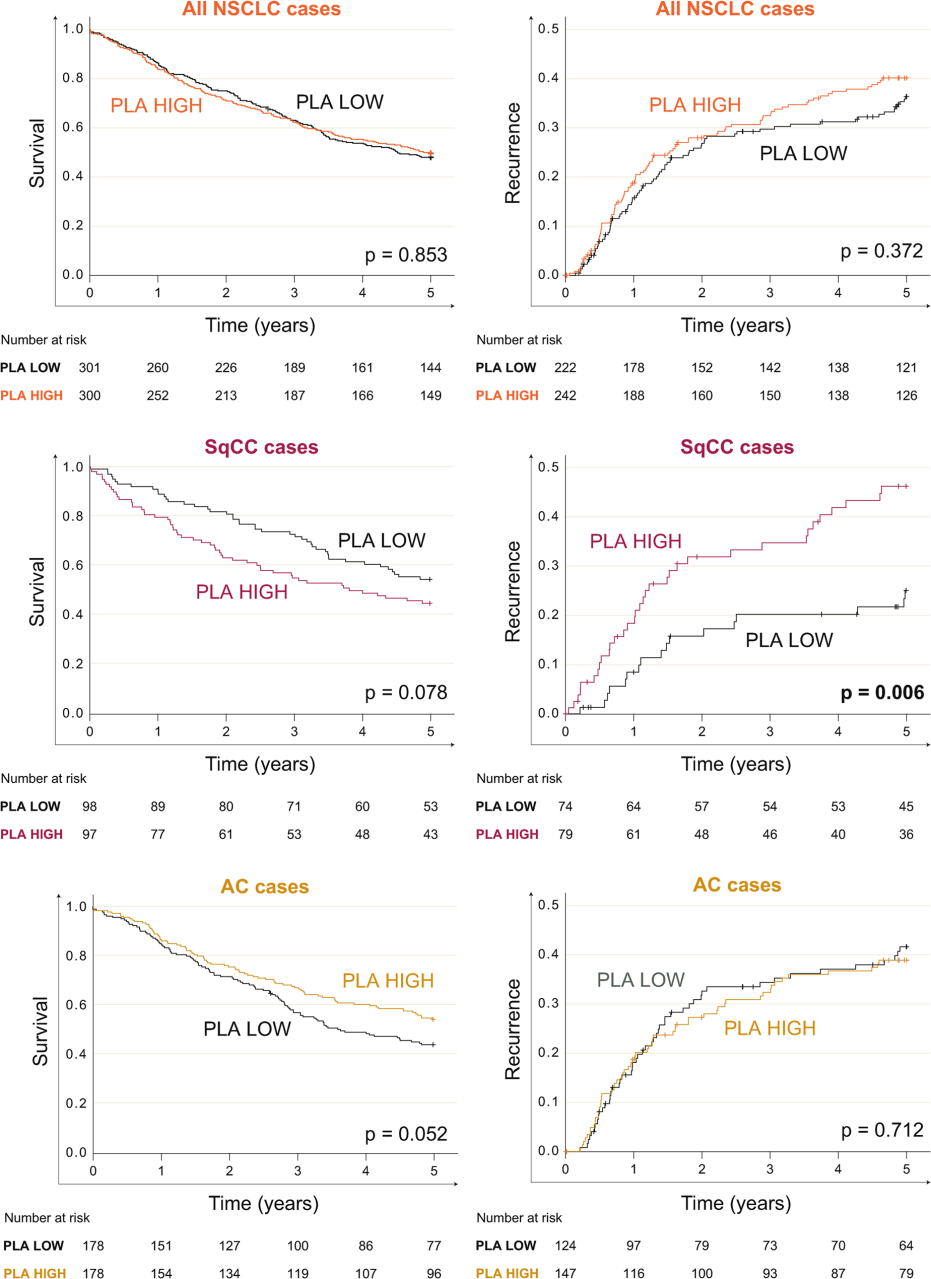
Grb2-PDGFRβ PLA activation score in relation to survival and recurrence. (Left column) Kaplan Meier curves demonstrating overall survival (truncated at 5 years) in all NSCLC patients (top), squamous cell carcinoma (SqCC) patients (middle), and adenocarcinoma (AC) patients (bottom). (Right column) Cumulative hazard plots for recurrence within 5 years of diagnosis for the same patient groups. Statistics are based on Log-rank tests. Low and high groups were defined by the median Grb2-PDGFRβ score as cut-point in each respective patient group separately

The dichotomized Grb2-PDGFRβ activation score (median cut-off) was not associated with overall survival in the complete NSCLC cohort, as displayed in a Kaplan Meier curve (Fig. 4A left, Log-rank test, *p* = 0.853) or in the Cox regression model (5-year survival, Hazard ratio univariable/HR_uni_=0.98[CI 0.78–1.23], *p* = 0.853). In subgroup analysis, SqCC patients with lower PDGFRβ activation score tended to survive longer compared to patients with high PDGFRβ activation scores (Fig. 4B left, HR_uni_=1.43 [CI: 0.96–2.12], *p* = 0.079). In contrast, in adenocarcinoma patients revealed a tendency towards longer survival with high PDGFRβ activation scores (Fig. 4C left, HR_uni_=0.75 [CI: 0.56-1.00], *p* = 0.053).

Similar associations were seen when the Grb2-PDGFRβ PLA score was associated with the 5-year recurrence rate (Fig. 4A-C right panel). Only in SqCC was a high activation score associated with increased recurrence risk (Log-rank test cumulative hazard plot, *p* = 0.006; Fig. 4B right; cause-specific HR_uni_=2.20 [CI: 1.23–3.95], *p* = 0.008). Importantly, the Grb2-PDGFRβ PLA score remained the only significant variable when tested in a cause-specific multivariable Cox regression model including also tumor stage, age, biological sex, and smoking history (HR_multi_ =2.31 [CI: 1.27–4.20], *p* = 0.006, Table 3). In line with this, the increased risk of recurrence in SqCC patients with a high PLA score was also reflected in a higher recurrence incidence compared to patients with a low score (43% *versus* 23%, Fisher’s exact test *p* = 0.010) (Supplementary Table 2, also statistically significant with the phosphoPDGFRβ assay). The site or organ of recurrence was documented, and the two most common locations -brain and lung- were examined for association with PDGFRβ activation scores; however, neither site showed a significant association with higher scores (Supplementary Table 2). No association with cancer recurrence was seen in the entire cohort or AC alone (Fig. 4A and C right), and the phosphoPDGFRβ PLA demonstrated the same survival and recurrence patterns (Supplementary Fig. 4).

**Table 3:** Multivariable hazard ratio (HR) for recurrence within 5 years post diagnosis. HRs are based on a cause-specific multivariable Cox regression model, treating recurrences as event and censoring death without prior recurrence. *P*-values are based on Wald’s test. Bold values indicate statistical significance

SqCC patients only	HR (95% CI)	*p*-value
PLA^#^ (LOW/HIGH)	2.31 (1.27–4.20)	**0.006**
Age at diagnosis (< 70/≥70 years)	0.91 (0.51–1.61)	0.736
Sex (male/female)	1.03 (0.57–1.86)	0.922
Smoking history (ever-/never-smoker)	0.83 (0.25–2.77)	0.757
Stage (I-II/III-IV)	1.54 (0.81–2.93)	0.187

#As via the Grb2-PDGFRβ assay

Finally, PDGFRβ protein levels assessed by IHC showed no association with overall survival or recurrence risk, whether analyzed across the entire cohort or within the SqCC and AC subgroups separately, using the subgroup-specific median as a cut-off (all *p* > 0.05, Supplementary Fig. 5).

### Associations between stromal PDGFRβ activation status and CAF and immune cell markers

Next, associations between the Grb2-PDGFRβ score and tumor microenvironmental features were examined, including immune cells as investigated with multiplexed immunofluorescence (mIF) as described previously [37], and vascularization and mesenchymal cell densities as investigated via IHC, using Spearman’s rank correlations (Supplementary Table 3). The stroma ratio, defined as the CK-negative area relative to CK-positive area, showed a very weak correlation with high PDGFRβ activation (Rho = 0.12, adj. p-value = 0.018). Among the stroma-infiltrating immune cell subsets analyzed, only cells with high expression of the Interleukin-3 receptor (CD123), likely representing plasmacytoid dendritic cells (pDCs) as defined by Backman et al. [37], showed a statistically significant, yet negative association with PDGFRβ activation (Rho=-0.20, adj. p-value = 0.013). Finally, the Grb2-PDGFRβ score correlated modestly with LRRC15, a known CAF marker [38] (Rho = 0.22, adj. p-value = 0.003); this association was, however, entirely driven by SqCC cases (Rho = 0.33, adj. p-value = 0.011), in which FAP also showed a positive correlation (Rho = 0.28, adj. p-value = 0.034).

## Discussion

Preclinical studies have demonstrated that PDGF signaling is a key regulator of fibroblasts and perivascular cells during tumor progression, prompting intensive exploration of PDGFRs as potential diagnostic biomarkers or attractive therapeutic targets across various solid tumors. Here, we present a novel in situ PLA strategy to reliably determine active stromal PDGFRβ signaling in diagnostic patient samples. By detecting specific protein–protein interactions or receptor phosphorylation events, this functional readout captures signaling activity that is more biologically relevant than receptor expression alone. Our results in this well-characterized NSCLC cohort demonstrate that measuring PDGFRβ activation provides refined information on tumor biology and patient prognosis compared to static expression levels. The applied PLA technique, which depicts specific protein interactions, may serve as a tool for a paradigm shift in clinical diagnostics, facilitating the prediction of therapy responses.

Current diagnostic approaches in personalized medicine rely on immunohistochemical stainings or molecular analyses of patient biopsies with the aim of improving diagnostic accuracy, prognostication, and stratifying patients for optimal treatment. Unfortunately, most diagnostic strategies are limited by FFPE fixation and minimal tissue availability. Conceptually, even more problematic is the fact that current assays only provide static information, i.e., whether a protein is expressed (IHC) or a gene is mutated (sequencing), but not whether these changes reflect pathway activity. This might explain the limited accuracy in predicting therapeutic effects for targeted therapy. Our concept fills that gap by directly mapping in situ PDGFRβ signaling events in NSCLC and is adaptable to other signaling pathways and cancer types.

The PLA enables the detection of two antigens in close proximity and can therefore map signal pathway activation, prototypically illustrated in our study via (1) Grb2-PDGFRβ interactions and (2) PDGFRβ tyrosine phosphorylations. Our thorough validation, including in vitro experiments with PDGF-BB stimulated cells, demonstrated the specificity of the assays. The validity of our assays is also highlighted by the fact that the Grb2-PDGFRβ and phosphoPDGFRβ PLAs delivered comparable data, demonstrated by the assays’ strong correlation.

This observation also supports the notion that protein phosphorylation is retained to a large extent even in FFPE tissues. Nevertheless, it should be considered that the Grb2-PDGFRβ PLA delivered an overall considerably higher signal density than the phosphorylation assay, possibly due to higher stability of protein-protein interactions in FFPE tissues, and thus might be superior to the phosphoPDGFRβ PLA concerning sensitivity. On the other hand, targeting PDGFRβ activation with only Grb2 may not accurately reflect the full downstream signaling activation pattern of the receptor. Several site-specific tyrosine phosphorylations of the intracellular domain of PDGFRβ lead to the binding or activation of Grb2, Grb7, Proto-oncogene c-Src (Src), Phospholipase C gamma (PLCγ) or Phosphoinositide 3-kinase (PI3K) among other signaling molecules [1–6], and their relative activation most likely differs in different cell types and under the influence of different cellular cues depending on the tumor microenvironment. Consequently, the Grb2-PDGFRβ interaction does not allow discrimination between the different downstream signaling pathways.

Given the general high interest in gaining a better understanding of PDGFRβ downstream signaling from the direct tissue context, a multiplex PLA approach on a single tissue section, targeting several downstream protein interactions or phosphorylations, appears to be an attractive molecular tool. Such a multiplex PLA would open the possibility of studying cellular signaling interactomes in situ. Still, as the Grb2-PDGFRβ strongly correlated with the overall PDGFRβ phosphorylation status, we believe it is a reliable surrogate assay for general PDGFRβ activation.

Elevated PDGFRβ activation scores in NSCLC were most pronounced when high receptor expression coincided with abundant PDGF ligand gene expression (*PDGFB*/*PDGFD*), highlighting ligand availability as a critical modulator of PDGFRβ signaling. Consequently, both PLAs distinguished NSCLC tumors with high stromal receptor expression and signaling activation from those with low activity. Thus, this functional readout results in a different NSCLC patient stratification. In our study, a high PDGFRβ activation status identified a subgroup of SqCC patients with an unfavorable prognosis and a higher risk of relapse, which was not detected with conventional PDGFRβ IHC. In contrast, no corresponding prognostic relationship was observed for AC patients, the most common histological subtype of NSCLC, nor the combined cohort, including also large cell carcinoma patients and other rare histologies, indicating that the prognostic relevance of stromal PDGFRβ activation appears specific to SqCC. Notably, previous studies assessing general stromal PDGFRβ levels in NSCLC have largely reported non-significant trends towards poorer survival, most consistently in AC [22, 27, 39]. One recent study analyzing the TCGA lung squamous cell carcinoma dataset reported an association between higher bulk *PDGFRB* mRNA levels and worse prognosis in this subtype, while comparable analyses in adenocarcinoma were not conducted [40]. Our analyses suggest that direct assessment of active PDGFRβ signaling provides more refined clinical information than general expression-based approaches alone. Nevertheless, tissue complexity and cellular heterogeneity may still attenuate the strength of our observations.

Indeed, PDGFRβ is expressed on multiple stromal cell populations, including fibroblast subsets, pericytes, and smooth muscle cells, and is occasionally detected in tumor cells as well [24]. Because PDGFRβ signaling effects are likely cell-type specific, future work should include cell type markers in parallel. This can likewise be achieved in a multiplexed assay that connects the PDGFRβ activation PLA not only with an epithelial cell marker, as in our study, but also with several multiplexed stroma cell markers (e.g., CD31, FAP, CD45). Such an approach would result in multiple cell-type- and location-specific PDGFRβ activation scores. Given the strong preclinical evidence that PDGFRβ signaling regulates tumor vascularization and drug penetration, it is plausible that clinically relevant effects of active PDGFRβ signaling would emerge in a therapeutic context, e.g., with antiangiogenic therapies. Notably, Sunitinib, a multi-targeted RTK inhibitor with high affinity for VEGFRs, c-KIT, and PDGFRs, is approved for advanced renal-cell carcinoma, gastrointestinal stromal tumors, and pancreatic neuroendocrine tumors, although only a minority of patients experience benefit. It could be speculated whether a PDGFRβ activation assay as a companion diagnostic would be of value to predict responses in these patients. This example illustrates just one application of the diagnostic potential of functional assays, particularly for targeted therapy. In routine diagnostics, a brightfield-based PLA for PDGFRβ activation without CK staining could represent a feasible alternative in settings where the stromal compartment can be reliably identified morphologically during pathological assessment. However, irrespective of assay design, future studies will also need to systematically address threshold definition and optimization, especially for potential clinical implementation, where robust and reproducible cut-offs are essential.

Besides the clinical impact of the SqCC histological subtype, the PLA also revealed distinct biological stromal characteristics. PDGFRβ activation was associated with a higher stroma fraction and enrichment of LRRC15- and FAP-positive cells, suggesting a subtype-specific stromal phenotype that may contribute to the adverse outcome. This stroma phenotype may not be surprising, as PDGFRβ signaling reshapes the tumor microenvironment and is strongly linked to CAF phenotypes. Generally, PDGFRβ signaling mediates the proliferative aspect of CAFs, while LRRC15 and FAP are considered to define fibroblast subsets with immunosuppressive properties and poor prognosis [39, 41]. The distinct stromal characteristics of PDGFRβ-activated stroma are also reflected in the immune landscape, with a reduction of CD123-positive immune cells, likely pDCs [37]. pDCs are potent producers of type 1 interferons upon sensing nucleic acids, thereby aiding in antiviral defenses [42]. In cancer, however, they can exhibit both tumor-promoting [43] and tumor-suppressing effects depending on the tumor context, while their exact role remains unclear. Besides the reduction of CD123-positive immune cells in PDGFRβ-activated stroma, we also observe a tendency to increased regulatory T-cell infiltration, although multiple testing impairs the interpretation of these descriptive data. Of note, none of the observed immune cell associations remained statistically significant when analyses were performed within the individual histological subtypes. Further focused studies are clearly warranted to understand the complex interplay between CAFs, immune cells, and the stroma context in modulating the effects of PDGFRβ signaling.

In conclusion, our study presents a robust and specific tool to map PDGFRβ activation in diagnostic FFPE tissue sections. It provides clinically relevant information in early-stage surgically treated SqCC, beyond static protein expression. The applied PLA technique, which depicts specific protein interactions or protein modifications, may serve as a tool for a paradigm shift in clinical diagnostics, facilitating patient stratification for targeted therapy in the era of precision medicine.

## Supplementary Information

Supplementary Material 1.

## Data Availability

No datasets were generated or analysed during the current study.
