# Supplementary material for: In situ mapping of activated PDGFRβ defines a prognostic discrepancy between histological subtypes of NSCLC
Source: Cell Commun Signal. 2026 Jan 15;24:55. doi: 10.1186/s12964-026-02651-3 (PMC12849303; doi:10.1186/s12964-026-02651-3)
Supplement: Supplementary file 1 — Supplementary Material 1. [file 12964_2026_2651_MOESM2_ESM.pdf]

**In Situ Mapping of Activated PDGFRβ Defines a Prognostic Discrepancy Between Histological Subtypes of NSCLC.**

Amanda Lindberg, Louise Hellberg, Anaïs Grandon, Hui Yu, Viktoria Thurfjell, Erik Wåhlén, Neda Hekmati, Max Backman, Axel Cederholm, Artur Mezheyeuski, Anna Klemm, Johan Botling, Agata Zieba Wicher, Patrick Micke, Carina Strell


| | Page number |
|---|---|
| Supplementary Figure 1 | 2 |
| Supplementary Figure 2 | 4 |
| Supplementary Figure 3 | 6 |
| Supplementary Figure 4 | 8 |
| Supplementary Figure 5 | 10 |
| Supplementary Table 1 | 12 |
| Supplementary Table 2 | 13 |
| Supplementary Table 3 | 14 |

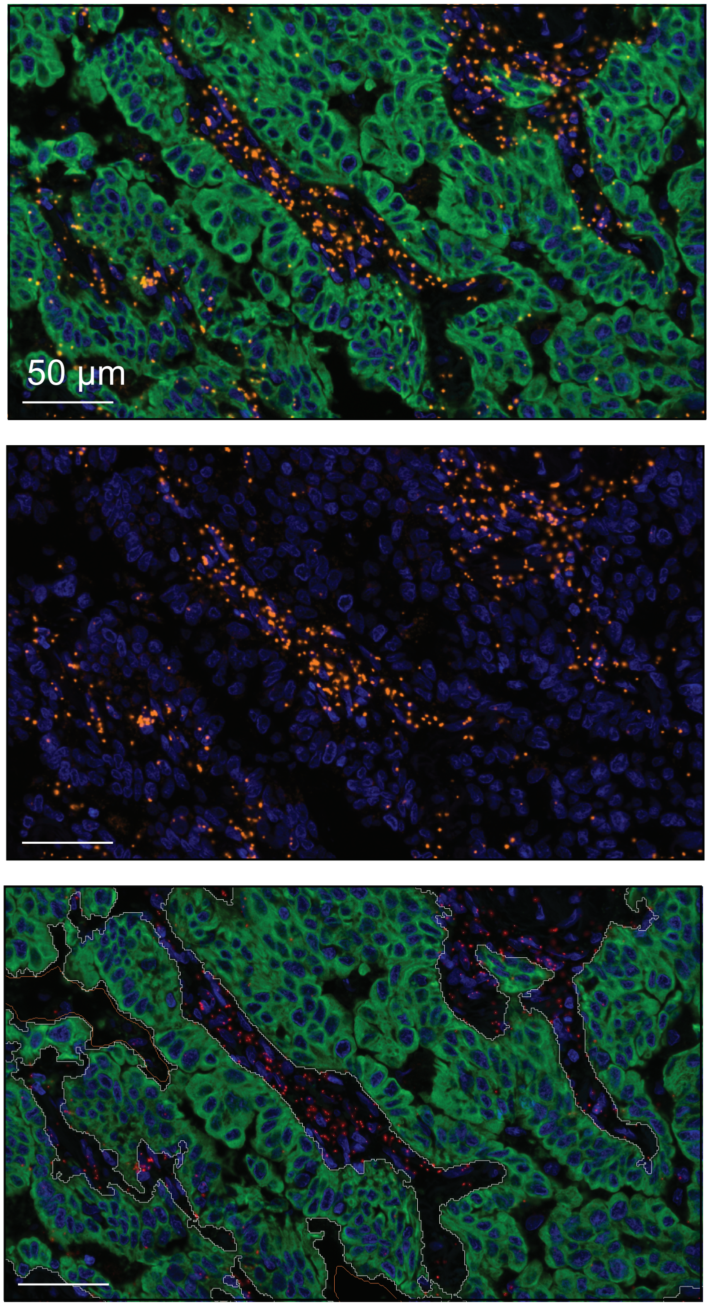
50 µm

**Supplementary Figure 1. Representative immunofluorescence staining and digital annotation of Grb2-PDGFRβ proximity ligation assay (PLA) signals in relation to cytokeratin (CK) expression.** The top panel shows the triplex immunofluorescence image with CK staining (green), Grb2-PDGFRβ PLA signals (orange), and nuclei counterstained with DAPI (blue). The middle panel displays the same field of view with the CK channel omitted, highlighting the distribution of PLA signals across both tumor (CK-positive) and stromal regions. The bottom panel illustrates the digitally generated annotation masks used for image analysis (orange contours), with tumor regions delineated by white contours and PLA signals identified specifically within the stromal compartment indicated by white pixel dots.

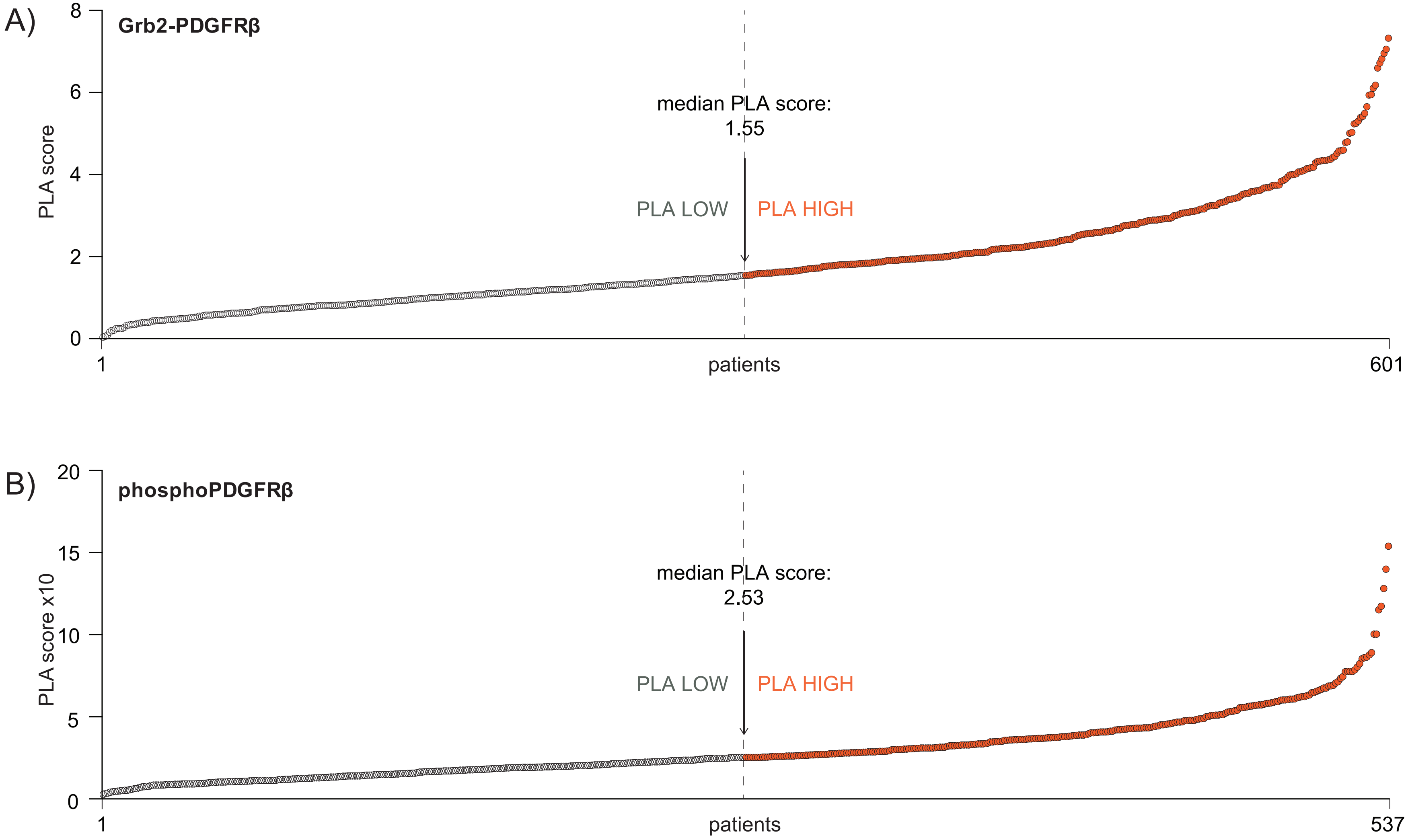
A)
Grb2-PDGFRβ
PLA score
8
6
4
2
0
median PLA score:
1.55
PLA LOW
PLA HIGH
1
patients
601
B)
phosphoPDGFRβ
PLA score x10
20
15
10
5
0
median PLA score:
2.53
PLA LOW
PLA HIGH
1
patients
537

**Supplementary Figure 2. Frequency distributions of PLA activation scores in the NSCLC patient cohort. A)** Distribution of the Grb2–PDGFRβ activation score among the 601 patients with successful staining and analysis. The arrow indicates the median score (1.55 dots per stromal cell). **B)** Distribution of the phosphoPDGFRβ activation score (displayed ×10) among the 537 patients with successful staining and analysis. The arrow indicates the median score (2.53 dots per stromal cell).

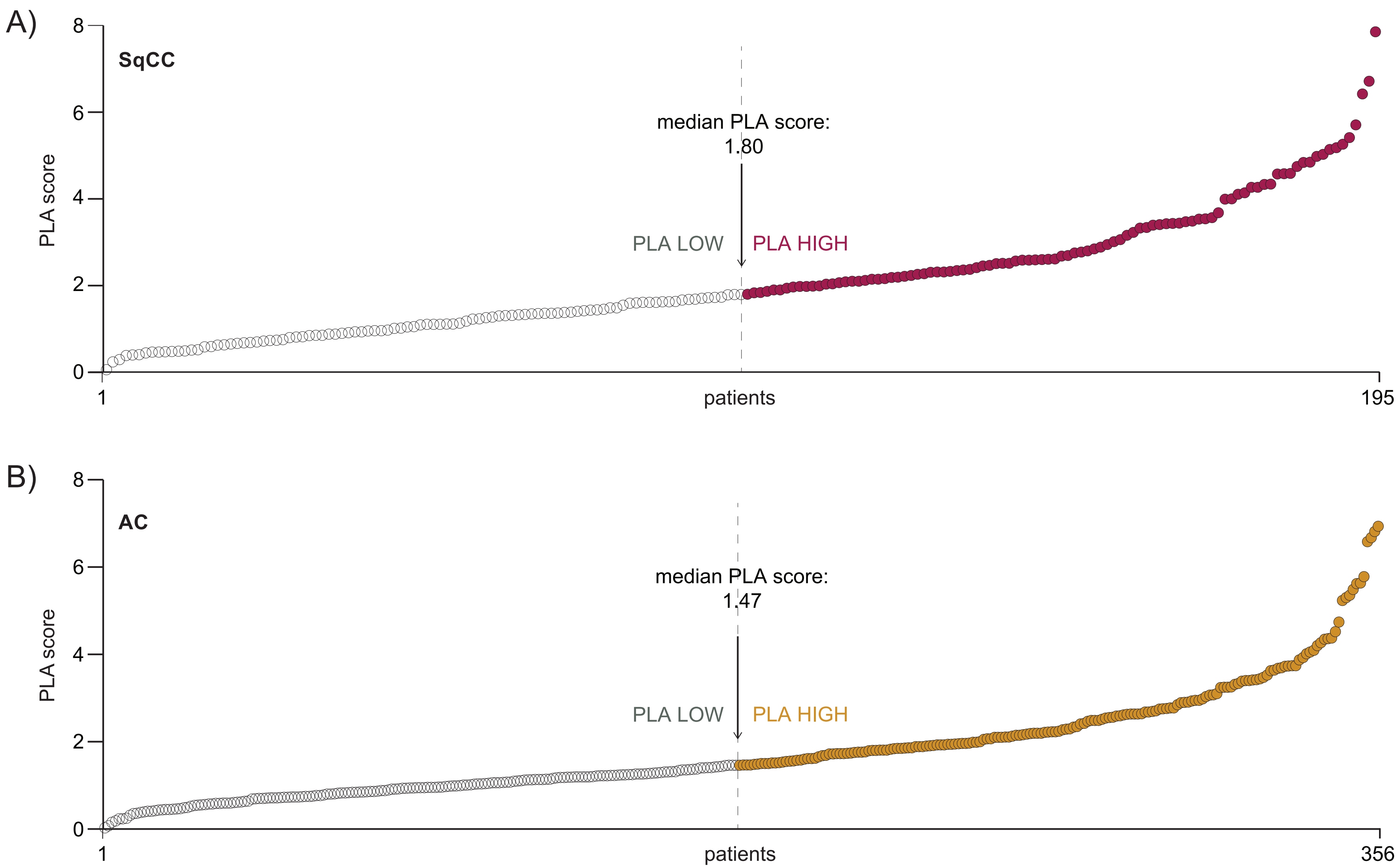
A)
SqCC
PLA score
8
6
4
2
0
median PLA score:
1.80
PLA LOW
PLA HIGH
1
patients
195
B)
AC
PLA score
8
6
4
2
0
median PLA score:
1.47
PLA LOW
PLA HIGH
1
patients
356

**Supplementary Figure 3. Frequency distributions of Grb2–PDGFRβ PLA activation scores across the two most common NSCLC histological subtypes separately. A)** Distribution of the Grb2–PDGFRβ activation score among the 195 squamous cell carcinoma (SqCC) patients with successful staining and analysis. The arrow indicates the median score (1.80 dots per stromal cell). **B)** Distribution of the Grb2–PDGFRβ activation score among the 356 adenocarcinoma (AC) patients with successful staining and analysis. The arrow indicates the median score (1.47 dots per stromal cell).

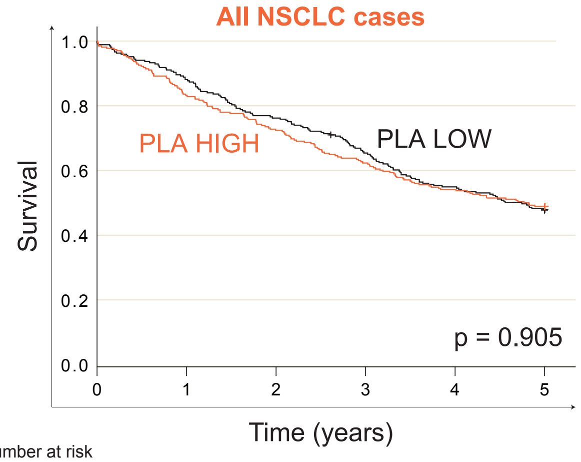


Number at risk

| | | | | | | |
|---|---|---|---|---|---|---|
| **PLA LOW** | 269 | 237 | 205 | 175 | 147 | 128 |
| **PLA HIGH** | 268 | 223 | 194 | 167 | 145 | 131 |

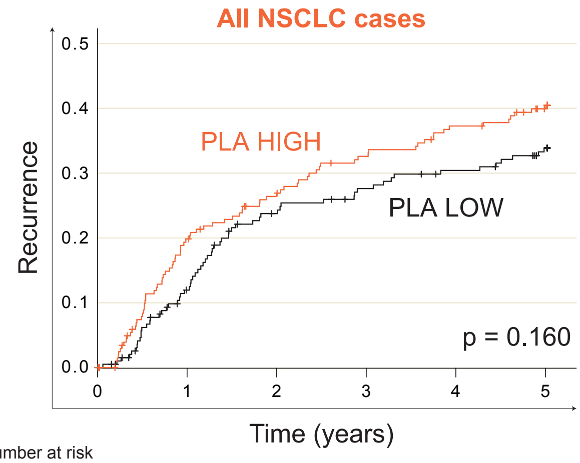


Number at risk

| | | | | | | |
|---|---|---|---|---|---|---|
| **PLA LOW** | 199 | 165 | 139 | 130 | 123 | 112 |
| **PLA HIGH** | 209 | 161 | 142 | 130 | 120 | 107 |

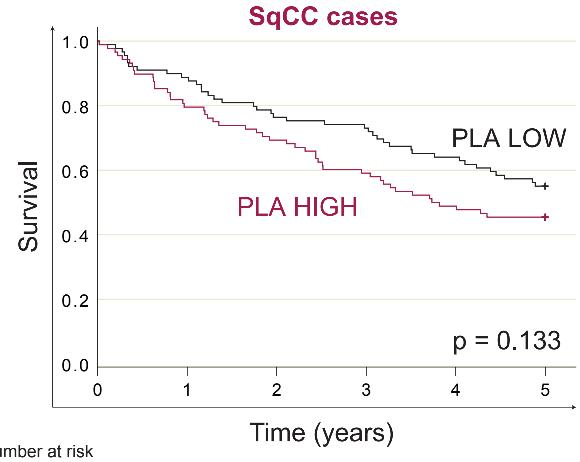


Number at risk

| | | | | | | |
|---|---|---|---|---|---|---|
| **PLA LOW** | 89 | 79 | 68 | 65 | 57 | 49 |
| **PLA HIGH** | 88 | 70 | 61 | 52 | 43 | 40 |

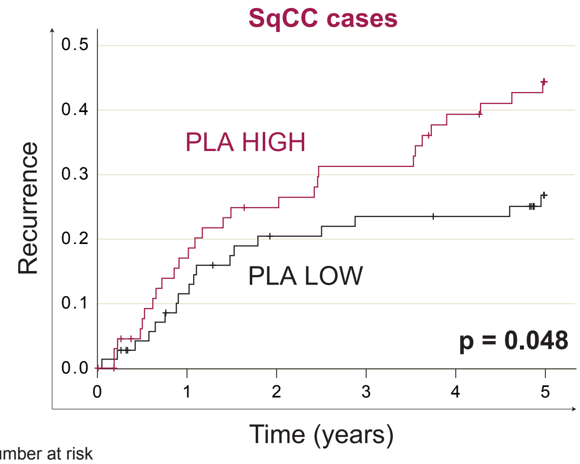


Number at risk

| | | | | | | |
|---|---|---|---|---|---|---|
| **PLA LOW** | 72 | 60 | 52 | 50 | 49 | 42 |
| **PLA HIGH** | 68 | 53 | 47 | 43 | 37 | 33 |

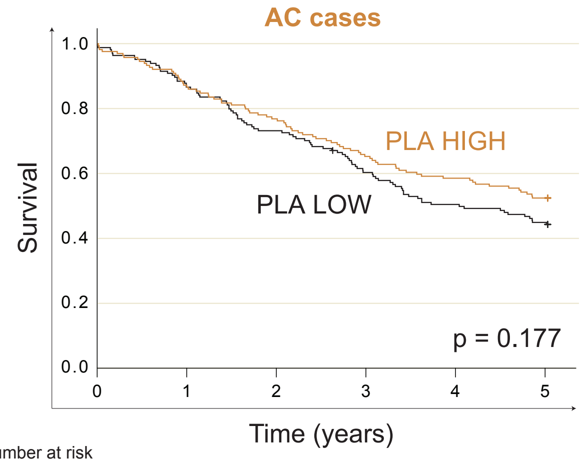


Number at risk

| | | | | | | |
|---|---|---|---|---|---|---|
| **PLA LOW** | 164 | 142 | 120 | 98 | 82 | 72 |
| **PLA HIGH** | 164 | 142 | 125 | 107 | 96 | 86 |

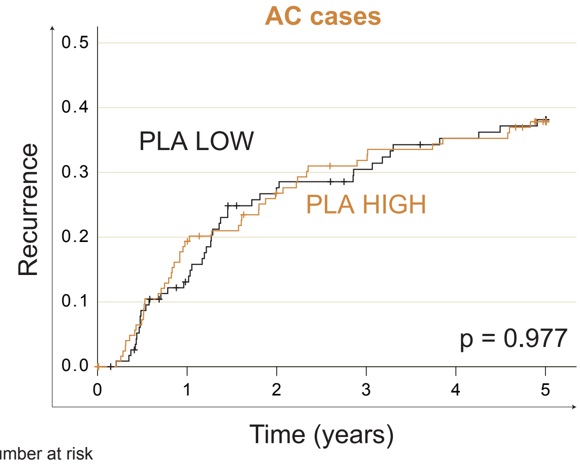


Number at risk

| | | | | | | |
|---|---|---|---|---|---|---|
| **PLA LOW** | 117 | 96 | 79 | 73 | 67 | 64 |
| **PLA HIGH** | 127 | 100 | 87 | 80 | 76 | 68 |

**Supplementary Figure 4. PhosphoPDGFRβ PLA activation score in relation to survival and recurrence.** (Left column) Kaplan Meier curves demonstrating overall survival (truncated at 5 years) in all NSCLC patients (top), squamous cell carcinoma (SqCC) patients (middle), and adenocarcinoma (AC) patients (bottom). (Right column) Cumulative hazard plots for recurrence within 5 years of diagnosis for the same patient groups. Statistics are based on Log-rank tests. Low and high groups were defined using the median phosphoPDGFRβ score as a cut-point in each respective patient group separately.

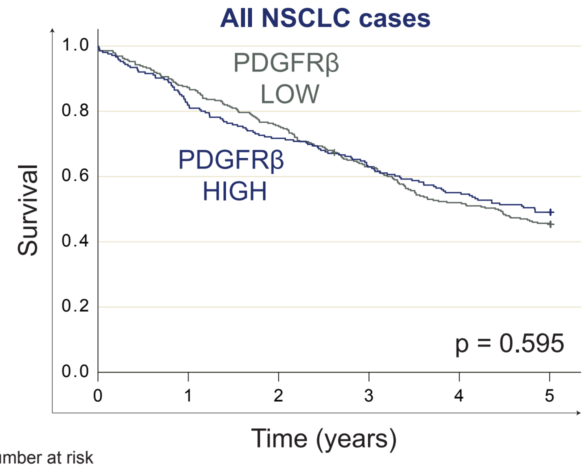


Number at risk

| | 0 | 1 | 2 | 3 | 4 | 5 |
|---|---|---|---|---|---|---|
| PDGFRβ LOW | 302 | 264 | 228 | 190 | 157 | 137 |
| PDGFRβ HIGH | 217 | 178 | 156 | 137 | 120 | 107 |

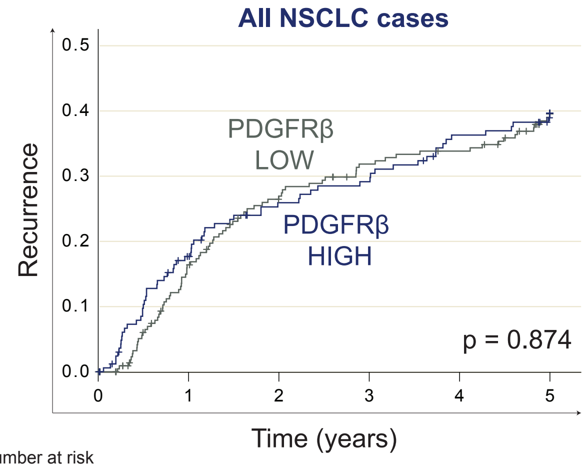


Number at risk

| | 0 | 1 | 2 | 3 | 4 | 5 |
|---|---|---|---|---|---|---|
| PDGFRβ LOW | 223 | 177 | 150 | 137 | 132 | 113 |
| PDGFRβ HIGH | 169 | 132 | 115 | 110 | 97 | 89 |

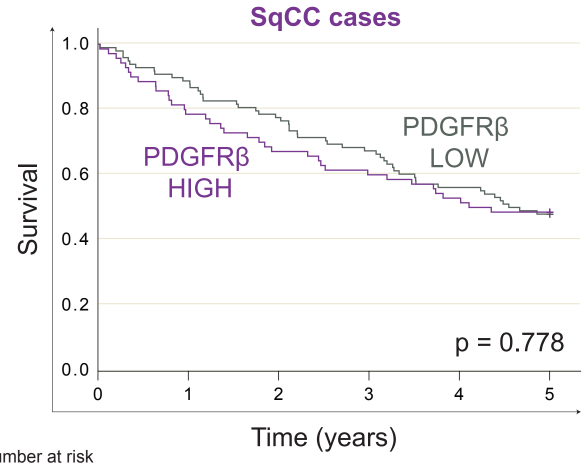


Number at risk

| | 0 | 1 | 2 | 3 | 4 | 5 |
|---|---|---|---|---|---|---|
| PDGFRβ LOW | 98 | 87 | 76 | 66 | 55 | 47 |
| PDGFRβ HIGH | 70 | 55 | 47 | 42 | 37 | 34 |

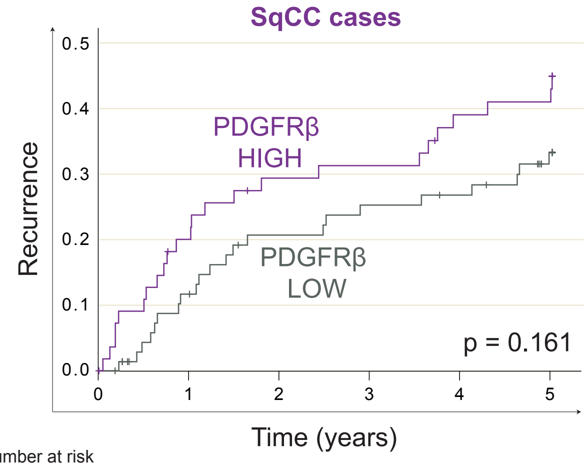


Number at risk

| | 0 | 1 | 2 | 3 | 4 | 5 |
|---|---|---|---|---|---|---|
| PDGFRβ LOW | 73 | 60 | 52 | 49 | 47 | 38 |
| PDGFRβ HIGH | 56 | 43 | 37 | 36 | 31 | 29 |

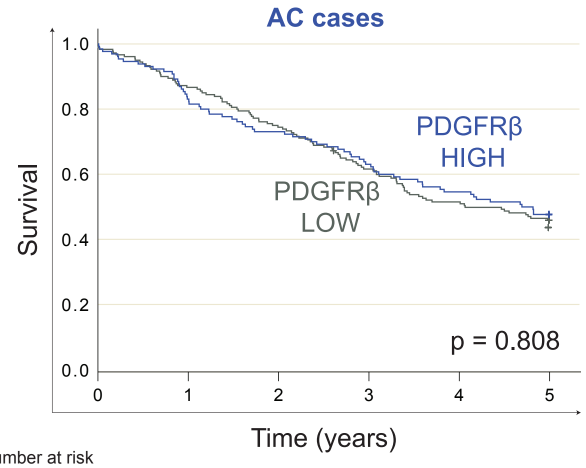


Number at risk

| | 0 | 1 | 2 | 3 | 4 | 5 |
|---|---|---|---|---|---|---|
| PDGFRβ LOW | 180 | 156 | 134 | 110 | 92 | 82 |
| PDGFRβ HIGH | 130 | 108 | 95 | 82 | 71 | 62 |

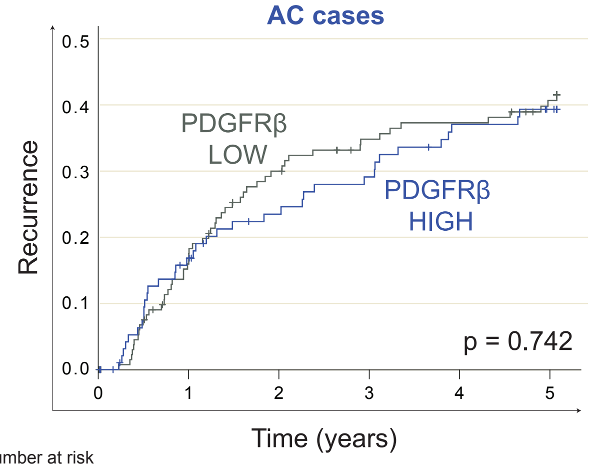


Number at risk

| | 0 | 1 | 2 | 3 | 4 | 5 |
|---|---|---|---|---|---|---|
| PDGFRβ LOW | 135 | 106 | 87 | 79 | 76 | 69 |
| PDGFRβ HIGH | 99 | 77 | 67 | 63 | 55 | 49 |

**Supplementary Figure 5. PDGFRβ protein expression level in relation to survival and recurrence.** (Left column) Kaplan Meier curves demonstrating overall survival (truncated at 5 years) in all NSCLC patients (top), squamous cell carcinoma (SqCC) patients (middle), and adenocarcinoma (AC) patients (bottom). (Right column) Cumulative hazard plots for recurrence within 5 years of diagnosis in the same patient groups. Statistics are based on Log-rank tests. Statistics are based on Log-rank tests. Low and high groups were defined using the median PDGFRβ protein expression level as cut-point, which was value=7 for all groups, based on semiquantitative intensity scoring (see Materials and Methods for annotation strategy).

**Supplementary Table 1.** Comparison of the distribution of clinicopathological characteristics between the phosphoPDGFRβ PLA LOW and PLA HIGH groups. P-values are based on Fisher's Exact tests for comparisons between 2 variables, and Fisher-Freeman-Halton Exact tests for comparisons between >2 variables. All tests were 2-sided.

| n (Valid %) | | | | | | | | | |
|---|---|---|---|---|---|---|---|---|---|
| | **All NSCLC patients n = 537** | | | **SqCC patients only n = 177** | | | **AC patients only n = 328** | | |
| **Variable** | **PLA LOW** n = 269 (50.1) | **PLA HIGH** n = 268 (49.9) | **p-value** | **PLA LOW** n = 89 (50.3) | **PLA HIGH** n = 88 (49.7) | **p-value** | **PLA LOW** n = 164 (50.0) | **PLA HIGH** n = 164 (50.0) | **p-value** |
| **Histology:** | | | | | | | | | |
| SqCC | 82 (30.5) | 95 (35.4) | 0.451 | - | - | - | - | - | - |
| AC | 171 (63.6) | 157 (58.6) | | - | - | | - | - | |
| Other | 16 (5.9) | 16 (6.0) | | - | - | | - | - | |
| **Stage 8th edition:** | | | | | | | | | |
| I | 144 (53.5) | 126 (47.0) | 0.369 | 43 (48.3) | 34 (38.6) | 0.427 | 91 (55.5) | 87 (53.0) | 0.842 |
| II | 67 (24.9) | 78 (29.1) | | 26 (29.2) | 31 (35.2) | | 36 (22.0) | 40 (24.4) | |
| III | 53 (19.7) | 61 (22.8) | | 20 (22.5) | 23 (26.1) | | 32 (19.5) | 34 (20.7) | |
| IV | 5 (1.9) | 3 (1.1) | | 0 (0.0) | 0 (0.0) | | 5 (3.0) | 3 (1.8) | |
| **Age at diagnosis:** | | | | | | | | | |
| <70 years | 158 (58.7) | 159 (59.3) | 0.930 | 51 (57.3) | 49 (55.7) | 0.880 | 100 (61.0) | 96 (58.5) | 0.736 |
| ≥70 years | 111 (41.3) | 109 (40.7) | | 38 (42.7) | 39 (44.3) | | 64 (39.0) | 68 (41.5) | |
| **Biological sex:** | | | | | | | | | |
| Male | 128 (47.6) | 138 (51.5) | 0.389 | 51 (57.3) | 61 (69.3) | 0.119 | 67 (40.9) | 73 (44.5) | 0.577 |
| Female | 141 (52.4) | 130 (48.5) | | 38 (42.7) | 27 (30.7) | | 97 (59.1) | 91 (55.5) | |
| **Smoking history:** | | | | | | | | | |
| Current/former smoker | 242 (90.0) | 237 (88.4) | 0.677 | 86 (96.6) | 83 (94.3) | 0.496 | 144 (87.8) | 141 (86.0) | 0.744 |
| Never smoker | 27 (10.0) | 31 (11.6) | | 3 (3.4) | 5 (5.7) | | 20 (12.2) | 23 (14.0) | |
| **Performance status ECOG:** | | | | | | | | | |
| 0 | 158 (58.7) | 156 (58.2) | 0.660 | 45 (50.6) | 45 (51.1) | 0.576 | 101 (61.6) | 106 (64.6) | 0.454 |
| 1 | 97 (36.1) | 102 (38.1) | | 41 (46.1) | 37 (42.0) | | 54 (32.9) | 53 (32.3) | |
| 2-4 | 14 (5.2) | 10 (3.7) | | 3 (3.4) | 6 (6.8) | | 9 (5.5) | 5 (3.0) | |
| ***KRAS*** [a]: | | | | | | | | | |
| WT | 197 (73.5) | 187 (69.8) | 0.388 | 87 (97.8) | 82 (93.2) | 0.168 | 110 (61.3) | 94 (57.3) | 0.500 |
| Mutated | 71 (26.5) | 81 (30.2) | | 2 (2.2) | 6 (6.8) | | 63 (38.7) | 70 (42.7) | |
| ***EGFR*** [a]: | | | | | | | | | |
| WT | 238 (88.8) | 240 (89.6) | 0.890 | 88 (98.9) | 88 (100.0) | 1.000 | 136 (83.4) | 136 (82.9) | 1.000 |
| Mutated | 30 (11.2) | 28 (10.4) | | 1 (1.1) | 0 (0.0) | | 27 (16.6) | 28 (17.1) | |
| ***TP53*** [b]: | | | | | | | | | |
| WT | 68 (39.8) | 67 (39.6) | 1.000 | 10 (17.2) | 7 (13.2) | 0.607 | 53 (51.0) | 59 (55.1) | 0.582 |
| Mutated | 103 (60.2) | 102 (60.4) | | 48 (82.8) | 46 (86.8) | | 51 (49.0) | 48 (44.9) | |

SqCC = Squamous cell carcinoma. AC = Adenocarcinoma. ECOG = Eastern Cooperative Oncology Group. *KRAS* = Kirsten rat sarcoma virus oncogene. *EGFR* = Epidermal growth factor receptor. *TP53* = Cellular Tumor Antigen p53. WT = Wildtype (genotype).
[a] = data missing for 1 patient. [b] = data missing for 197 patients.

**Supplementary Table 2.** Comparison of 5-year recurrence events between the Grb2-PDGFRβ PLA LOW and HIGH groups in squamous cell carcinoma (SqCC) patients. P-values are based on 2-sided Fisher's Exact tests.

| | n (Valid %) | | | | | |
|---|---|---|---|---|---|---|
| | SqCC patients only n = 195 | | | SqCC patients only n = 177 | | |
| | As via Grb2-PDGFRβ assay | | | As via phosphoPDGFRβ assay | | |
| **Variable** | **PLA LOW** n = 98 (50.3) | **PLA HIGH** n = 97 (49.7) | **p-value** | **PLA LOW** n = 89 (50.3) | **PLA HIGH** n = 88 (49.7) | **p-value** |
| **Recurrence at 5 years [a]:** | | | | | | |
| None | 57 (77.0) | 45 (57.0) | **0.010** | 56 (75.7) | 40 (58.8) | **0.048** |
| Yes | 17 (23.0) | 34 (43.0) | | 18 (24.3) | 28 (41.2) | |
| **Recurrence organ [b] (valid n=50/46):** | | | | | | |
| Brain | 1 (6.3) | 7 (20.6) | 0.409 | 3 (16.7) | 4 (14.3) | 1.000 |
| All other | 15 (93.8) | 27 (79.4) | | 15 (83.3) | 24 (85.7) | |
| **Recurrence site/organ [b] (valid n=50/46):** | | | | | | |
| Lung | 10 (62.5) | 11 (32.4) | 0.066 | 8 (44.4) | 12 (42.9) | 1.000 |
| All other | 6 (37.5) | 23 (67.6) | | 10 (55.6) | 16 (57.1) | |

SqCC = Squamous cell carcinoma. Sites/organs in which recurrences were reported: brain, lung, bones, liver, skin, mediastinum, adrenal glands, pleura, other.

[a] = data missing for 42 patients in the Grb2-PDGFRβ assay/data missing for 37 patients in the pPDGFRβ assay.

[b] = data is based on solely patients with recurrence – recurrence site/organ specified (brain or lung) can have additional recurrences.

**Supplementary Table 3.** Spearman's rank correlation coefficients between the Grb2-PDGFRβ PLA (continuous values) and other stromal factors previously assessed via multiplexed immunofluorescence or immunohistochemistry(37). P-values are two-sided and were adjusted for multiple testing using the Benjamini-Hochberg procedure for each patient set (all NSCLC patients, SqCC patients, AC patients) individually.

| | n (Valid %) | | | | | | | | |
|---|---|---|---|---|---|---|---|---|---|
| **Variable** | **All NSCLC patients** | **p-value** | **Adjusted p-value** | **SqCC patients only** | **p-value** | **Adjusted p-value** | **AC patients only** | **p-value** | **Adjusted p-value** |
| **Immune infiltration** | | | | | | | | | |
| B cell | 0.03 | 0.619 | 0.689 | -0.10 | 0.360 | 0.565 | 0.15 | 0.068 | 0.146 |
| CD4-effector | 0.10 | 0.119 | 0.257 | -0.01 | 0.923 | 0.923 | 0.16 | **0.044** | 0.140 |
| CD4-Treg | 0.13 | **0.043** | 0.133 | 0.10 | 0.368 | 0.565 | 0.16 | 0.049 | 0.140 |
| CD8-effector | 0 06 | 0.352 | 0.460 | -0.11 | 0.303 | 0.565 | 0.18 | **0.026** | 0.140 |
| CD8-Treg | 0.13 | **0.047** | 0.133 | 0.05 | 0.631 | 0.767 | 0.17 | **0.037** | 0.140 |
| CD163 | 0.08 | 0.192 | 0.363 | 0.12 | 0.303 | 0.565 | 0.11 | 0.186 | 0.316 |
| iDC | 0.08 | 0.247 | 0.370 | -0.05 | 0.632 | 0.767 | 0.13 | 0.132 | 0.249 |
| mDC | -0.03 | 0.648 | 0.689 | -0.09 | 0.399 | 0.565 | 0.05 | 0.524 | 0.631 |
| pDC | -0.20 | **0.002** | **0.013** | -0.27 | **0.015** | 0.082 | -0.17 | **0.047** | 0.140 |
| M1 macrophage | -0.04 | 0.532 | 0.646 | -0.13 | 0.232 | 0.565 | -0.05 | 0.557 | 0.631 |
| M2 macrophage | 0.07 | 0.254 | 0.370 | 0.04 | 0.732 | 0.777 | 0.11 | 0.205 | 0.317 |
| NK cell | 0.07 | 0.261 | 0.370 | 0.15 | 0.187 | 0.565 | 0.04 | 0.640 | 0.680 |
| NKT cell | -0.02 | 0.746 | 0.746 | -0.05 | 0.685 | 0.777 | -0.02 | 0.825 | 0.825 |
| **Stroma ratio** | | | | | | | | | |
| Stroma area (CK negative) | 0.12 | **0.003** | **0.018** | 0.11 | 0.119 | 0.508 | 0.13 | **0.014** | 0.140 |
| **Mesenchymal markers** | | | | | | | | | |
| LRRC15 | 0.22 | **<0.001** | **0.003** | 0.33 | **0.001** | **0.011** | 0.14 | 0.069 | 0.146 |
| FAP | 0.12 | **0.040** | 0.133 | 0.28 | **0.004** | **0.034** | 0.05 | 0.484 | 0.631 |
| CD31 | -0.09 | 0.121 | 0.257 | -0.12 | 0.241 | 0.565 | -0.06 | 0.449 | 0.631 |

SqCC = Squamous cell carcinoma. AC = Adenocarcinoma.
